# Biodiversity between sand grains: Meiofauna composition across southern and western Sweden assessed by metabarcoding

**DOI:** 10.3897/BDJ.8.e51813

**Published:** 2020-04-27

**Authors:** Sarah Atherton, Ulf Jondelius

**Affiliations:** 1 Swedish Museum of Natural History, Stockholm, Sweden Swedish Museum of Natural History Stockholm Sweden

**Keywords:** Illumina Mi-Seq, High Throughput Sequencing, Acoela, Gastrotricha, Platyhelminthes, Macrostomorpha, Rhabdocoela, Species Identification

## Abstract

The meiofauna is an important part of the marine ecosystem, but its composition and distribution patterns are relatively unexplored. Here we assessed the biodiversity and community structure of meiofauna from five locations on the Swedish western and southern coasts using a high-throughput DNA sequencing (metabarcoding) approach. The mitochondrial cytochrome oxidase 1 (COI) mini-barcode and nuclear 18S small ribosomal subunit (18S) V1-V2 region were amplified and sequenced using Illumina MiSeq technology. Our analyses revealed a higher number of species than previously found in other areas: thirteen samples comprising 6.5 dm^3^ sediment revealed 708 COI and 1,639 18S metazoan OTUs. Across all sites, the majority of the metazoan biodiversity was assigned to Arthropoda, Nematoda and Platyhelminthes. Alpha and beta diversity measurements showed that community composition differed significantly amongst sites. OTUs initially assigned to Acoela, Gastrotricha and the two Platyhelminthes sub-groups Macrostomorpha and Rhabdocoela were further investigated and assigned to species using a phylogeny-based taxonomy approach. Our results demonstrate that there is great potential for discovery of new meiofauna species even in some of the most extensively studied locations.

## Introduction

Meiofauna (i.e. animals that pass through a 1 mm mesh, but are retained on a 45 µm mesh; Higgins and Thiel 1988) have been relatively unexplored (Eisenhauer et al. 2019), despite the fact that they are an important component of benthic habitats and play key roles in their environments (Aller and Aller 1992, Schratzberger and Ingels 2018, Snelgrove and Smith 2002). Assessments of meiobenthic diversity have often been performed manually using traditional morphological approaches, whereby researchers pick individual animals from a sample and attempt to identify the animal to species level (Giere 2009, Higgins and Thiel 1988). Such explorations are time-consuming and require high levels of taxonomic expertise and are therefore, by-necessity, focused on a limited number of taxonomic groups. Moreover, there is potential for great amounts of missed diversity, as animals that are particularly small, delicate, or simply unusual may be missed or ignored, while the presence of asexual or juvenile animals could make identification impossible.

Nevertheless, there is a long history of studies on meiofaunal taxa in the North Sea and Baltic waters surrounding Sweden. For instance, Westblad (Westblad 1940, Westblad 1942, Westblad 1945, Westblad 1946, Westblad 1948) published a series of papers describing Acoela and Platyhelminthes from the Gullmarn Fjord and surrounding Swedish and Norwegian waters, while Swedish marine Rhabdocoela (Platyhelminthes) were assessed on several occasions by, for example, Karling (Karling 1956a, Karling 1956b, Karling 1963, Karling 1974), Luther (Luther 1962, Luther 1963) and Westblad (Westblad 1954). For a long time, the Kristineberg Marine laboratory was especially the focal point of many studies of meiofaunal diversity (e.g. Boaden 1960, Elofson 1944, Jägersten 1952, Odhner 1937 and many others) and broad meiofaunal surveys have more recently occurred at the the Sven Lovén Centre for Marine Sciences on the island of Tjärnö (Willems et al. 2009, Curini-Galletti et al. 2012) . As a consequence, the Swedish meiofauna is comparatively well known.

Further in 2002, the Swedish Species Information Centre (ArtDatabanken) established a 20-year project to improve the taxonomical knowledge of all Swedish multicellular organisms and new opportunities were provided for modern day surveys of many different meiofaunal groups, including such taxa-of-interest as Acoela (e.g. Kånneby et al. 2015), Gastrotricha (e.g. Kånneby et al. 2009, Kånneby et al. 2012, Kånneby 2011, Kånneby et al. 2013) and Platyhelminthes (e.g. Atherton and Jondelius 2018a, Atherton and Jondelius 2018b, Atherton and Jondelius 2019, Larsson et al. 2008, Larsson and Willems 2010). The intense sampling activities during these surveys resulted in discovery of species new to science and new records for Sweden. In addition, molecular barcoding sequences for both new and known species were attained and deposited in public databases. The availability of these and other DNA sequences allowed for the creation of reference databases, which are a critical component in the recently developed metabarcoding approach to biodiversity assessment.

High-throughput DNA sequencing of a subset of gene(s) extracted from environmental or bulk samples (i.e. metabarcoding; TABERLET et al. 2012) is a promising tool that allows for rapid and comprehensive examination of community compositions (Brannock and Halanych 2015, Leray and Knowlton 2016). Metabarcoding techniques have been previously used to assess metazoan colonies along autonomous reef monitoring structures (Leray and Knowlton 2015), planktonic assemblages (de Vargas et al. 2015, Lindeque et al. 2013) and marine benthic meiofaunal communities (Brannock and Halanych 2015, Cowart et al. 2015, Creer et al. 2010, Fonseca et al. 2014, Fonseca et al. 2010). Two prior studies used metabarcoding approaches to examine meiobenthic communities in Sweden (Haenel et al. 2017, Holovachov et al. 2017) with a primary focus on improving and testing methods of metabarcoding, including examining the effects of sediment extraction methods, the usefulness of different primer pairs and gene loci and the optimal strategies to assign and identify operational taxonomic units (OTUs).

In this study, five locations across the southern and western coast of Sweden were sampled and a high-throughput metabarcoding approach was employed to provide a snapshot of their meiobenthic communities using fragments of the mitochondrial COI and the nuclear 18S rDNA genes. How efficient was the metabarcoding approach in capturing previously-recorded species diversity? Will metabarcoding reveal hitherto undetected species, even in localities that have been extensively sampled with traditional methods? To address these questions, four meiofaunal groups previously surveyed as part of the Swedish Taxonomy Initiative and hence with available sequence reference databases were selected for further assessment and OTUs assigned to these groups were identified to species level.

## Material and methods

### Sampling

**Figure 1.**
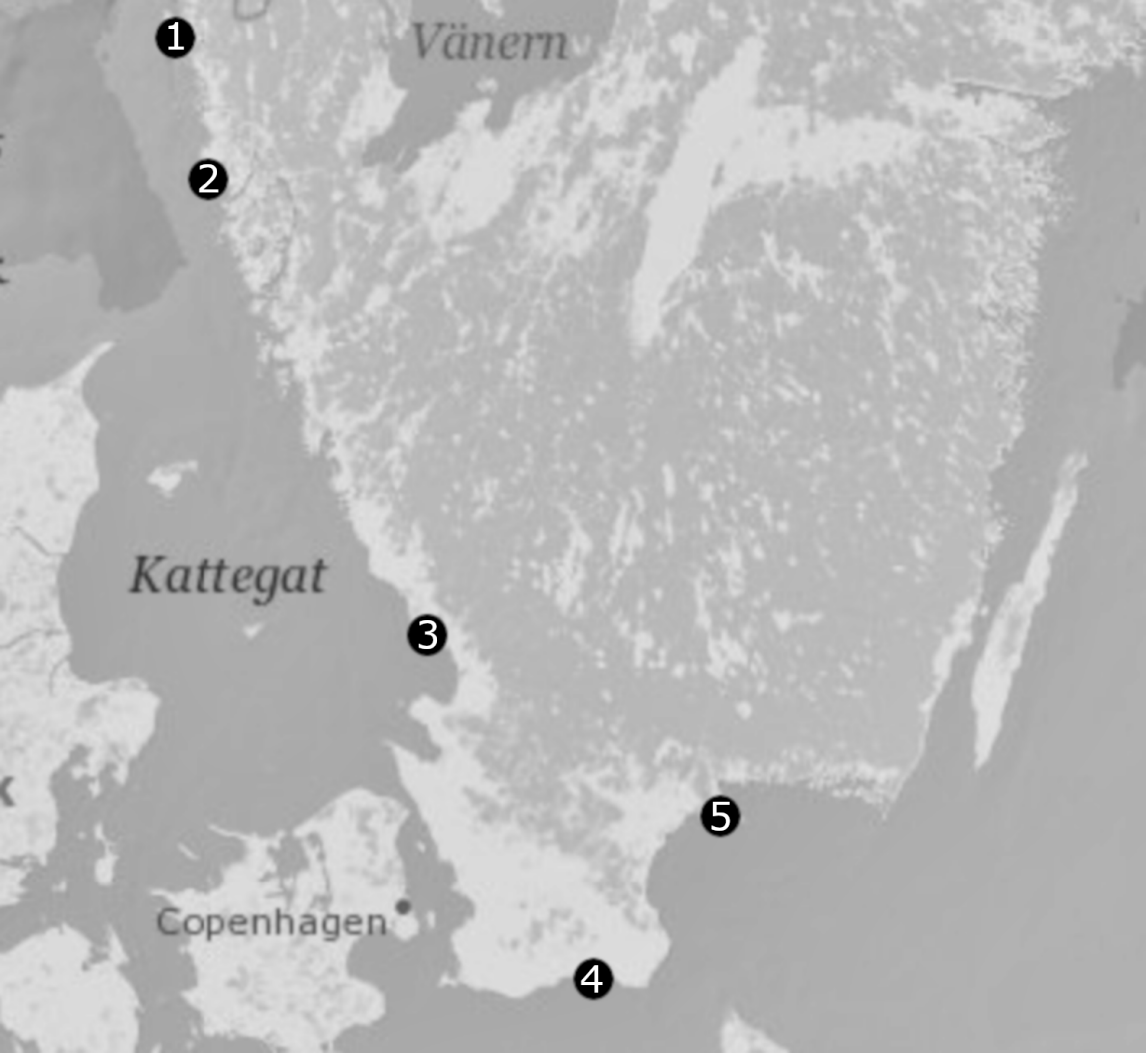
Location of each of the sampling sites along the Swedish western and southern coasts. (1) Tjärnö near the Tjärnö Marine Laboratory (formerly Sven Lovén Centre for Marine Sciences Tjärnö), 58°52’41.0 N, 11°06’56.0 E; (2) Fiskebäckskil near the Sven Lovén Centre for Marine Sciences Kristineberg, 58°14’52.1 N, 11°27’05.8 E; (3) Halmstad at Påarp Beach, 56°36’10.0 N, 12°54’48.3 E; (4) Kåseberga, just east of Ales stenar, 55°23’00.6 N, 14°03’46.5 E; (5) Landön western side, 55°58’20.6 N, 14°24’29.6 E.

Marine sandy sediments were collected from five locations within Sweden during May-June 2016 (Fig. 1). Sites 1 and 2 are situated near the Marine labs at Tjärnö and Kristineberg where, especially at site 2, there is a long history of taxonomic meiofauna studies (see Coull and Giere 1988, Swedmark 1964 on the history of meiofauna research). The remaining three sites span the Swedish southern coastline where little previous sampling of the meiofauna has occurred.

At each site, two or three replicates of 500 ml sediment were collected at 1.5 m depth, placed in jars and transported to the laboratory for processing. Sediments were placed in a 7.2% solution of MgCl_2_ initially for two minutes and then again for an additional five minutes. After both time periods, the samples were thoroughly mixed to suspend meiofauna and lighter sediment particles and the supernatant decanted through a 125 µm sieve. Samples were then fixed immediately in 95% ethanol for molecular analyses and stored at -20°C.

### Library preparation and sequencing

DNA was extracted using Qiagen’s DNeasy PowerSoil Kit following the manufacturer’s instructions.

Primers were selected from Haenel et al. (2017) to target a 370 bp fragment of the 18S gene corresponding to the V1-V2 region, which have been shown to be effective for the amplification and identification of metazoan diversity in previous metabarcoding studies (Haenel et al. 2017, Holovachov et al. 2017, Leasi et al. 2018). In addition, the COI reverse ‘Folmer’ primer (dgHCO2198, Folmer et al. 1994), as well as a newly-designed forward primer, were used to target a 390 bp fragment of the mitochondrial cytochrome oxidase 1 (CO1) gene encompassing the ‘mini-barcode’ region (Leray et al. 2013). Following the dual PCR amplification method of Bourlat et al. (2016), Illumina overhang adapter sequences were appended to the primers for the first PCR (the amplicon specific PCR), while a second PCR (the index PCR) was then performed to incorporate Illumina index adapters. Suppl. material 1 lists all primer information.

The amplicon PCR reactions were performed using 0.2 ml PuReTaq Ready-To-Go PCR Beads (GE Healthcare) with 5 pmol each forward and reverse primers and 3 µl DNA and cycling conditions of 5 min at 95°C, 35 cycles of (30s at 95°C, 90s at 50°C, 60s at 72°C) and 10 min at 70°C. PCR reactions were checked on a 2% agarose gel and purified with Agencourt AMPure XP magnetic beads (Beckman Coulter).

The index PCR reactions were performed using 0.2 ml PuReTaq Ready-To-Go PCR Beads (GE Healthcare) with 5 pmol each of Nextera XT Index Primer i5 and Nextera XT Index Primer i7 and 13 µl DNA. Cycling conditions consisted of 5 min at 95°C, 10 cycles of (30s at 95°C, 30s at 62°C, 30s at 72°C) and 10 min at 70°C. PCR reactions were again checked on a 2% agarose gel and purified with Agencourt AMPure XP magnetic beads (Beckman Coulter) using a 0.8 ratio to select for > 200bp fragments.

In order to minimise random sampling error, three libraries were created and run independently, each multiplexed to include amplicons from every sample. Libraries were pooled to equimolar concentration and sent to SciLifeLab (Stockholm, Sweden) for sequencing via the Illumina MiSeq platform with v3 chemistry. A total of 41,410,434 and 20,484,405 paired end reads of appropriate length were produced for 18S and CO1, respectively (Table 1).

### Bioinformatic processing

Bioinformatic data processing was performed via QIIME2 (Quantitative Insight Into Microbial Ecology) version 2018.11 (Caporaso et al. 2010) following the procedures presented in Bourlat et al. (2016). Sequence quality was initially assessed using FastQC (Andrews 2010), with primer removal, further quality control, chimera removal and OTU grouping performed via DADA2. Example scripts are presented in Suppl. material 2.

### Alpha and Beta diversity analyses

Overall species richness was calculated for each location in QIIME2 using the nonparametric Chao1 index (Chao 1984) with rarefied datasets to correct for bias due to unequal sampling size. One sample of the COI dataset with a very low yield (433 total sequences and 7 total OTUs) was excluded prior to all analyses. Rarefaction was performed without replacement to a depth of 141,711 for 18S sequences and 269,894 for COI sequences and was equal to the number of sequences in the smallest sample for each dataset. Alpha diversities amongst sites were compared by Analysis of Variance (ANOVA).

Principal coordinate analysis (PCoA) and Analysis of Similarities (ANOSIM) tests were performed to assess Beta diversity and differences in sample community composition. Distance matrices of the rarefied 18S and CO1 sample datasets were calculated in QIIME2 based on the Jaccard index (Jaccard 1901) and Analysis of Similarities (ANOSIM) tests were performed both pairwise and across all groups with 999 permutations. Example scripts for all alpha and beta diversity analyses are presented in Suppl. material 2.

### Preliminary OTU assignment

For 18S sequences, preliminary OTU taxonomy was assigned using QIIME’s feature-classifier classify-sklearn with SILVA release 128 at 99% (Quast et al. 2013) and default settings. This identifies query sequences to phyla, based on similarity levels of 80% and to species at 97%. A total of 281 OTUs were identified as sequences belonging to four, previously relatively well-studied taxa of interest (Acoela, Gastrotricha, Macrostomorpha and Rhabdocoela) and further processed for phylogeny-based taxonomic assignment.

COI sequences were blasted against the full NCBI nucleotide database (http://blast.nlm.nih.gov/Blast) and MEGAN v 6.17 (MEtaGenomics Analyzer; Huson et al. 2011) was used to parse the results and assign preliminary OTU taxonomy (min. support 1; min. score 100; top percent 10; min. complexity 0). As with the 18S OTUs, a total of 58 COI OTUs were identified as sequences of interest and processed for phylogeny-based taxonomic assignment.

### Phylogeny-based taxonomy assignments of taxa of interest

Reference alignments and phylogenetic trees were created in order to identify OTUs of the four taxa of interest and were constructed using 18S and COI gene sequences downloaded from GenBank. Datasets include 1) for Acoela, a total of 343 (18S) and 185 (COI) reference sequences, including a combination of sequences downloaded from GenBank, as well as new, previously unpublished sequences; 2) for Gastrotricha, 174 (18S) and 148 (COI) sequences, based on the combined dataset of Kånneby et al. 2013, Kieneke et al. 2012, Kolicka et al. 2018; 3) for Macrostomorpha, 239 (18S) and 95 (COI) sequences, based on Atherton and Jondelius 2019; and 4) for Rhabdocoela, 393 (18S) and 43 (COI) reference sequences representing all Rhabdocoela sequences currently available on GenBank. All reference alignments were carried out via MAFFT v 7 (https://mafft.cbrc.jp/alignment/server; Katoh et al. 2019) and the maximum likelihood (ML) trees were built using RAxML v. 8.2.10 (Stamatakis 2014) with a GTR-GAMMA substitution model and 1000 bootstrap replicates. Specimen information, including GenBank accession numbers, can be found in Suppl. material 3 .

Query sequences were aligned against the appropriate reference database using MOTHUR v 1.39 (Schloss et al. 2009) with default settings. Taxonomic predictions were generated via PPlacer v 1.1 (Matsen et al. 2010). Taxonomic assignments were based either on high likelihood (above 90% threshold) of single placement or on high cumulative likelihood of multiple placements when all are within a single monophyletic clade (see Holovachov et al. 2017). All reference alignments and Placement trees are available as Suppl. materials 4, 5, 6, 7, 8, 9, 10, 11, 12, 13, 14, 15, 16, 17, 18, 19.

## Results

### Overall community composition

Illumina MiSeq produced at total of 41,410,434 raw reads of 18S and 20,484,405 raw reads of CO1, which were reduced following the quality filter step to 9,691,423 and 9,043,200 reads, respectively (Table 1). Reads were clustered into a total of 3,615 18S and 2,276 COI representative OTUs and an 80% similarity threshold was used to determine phylum level taxonomy. All MiSeq data underpinning the analyses reported in this paper are deposited at the GenBank SRA under project number PRJNA627723 (https://www.ncbi.nlm.nih.gov/bioproject/PRJNA627723).

**Figure 2.**
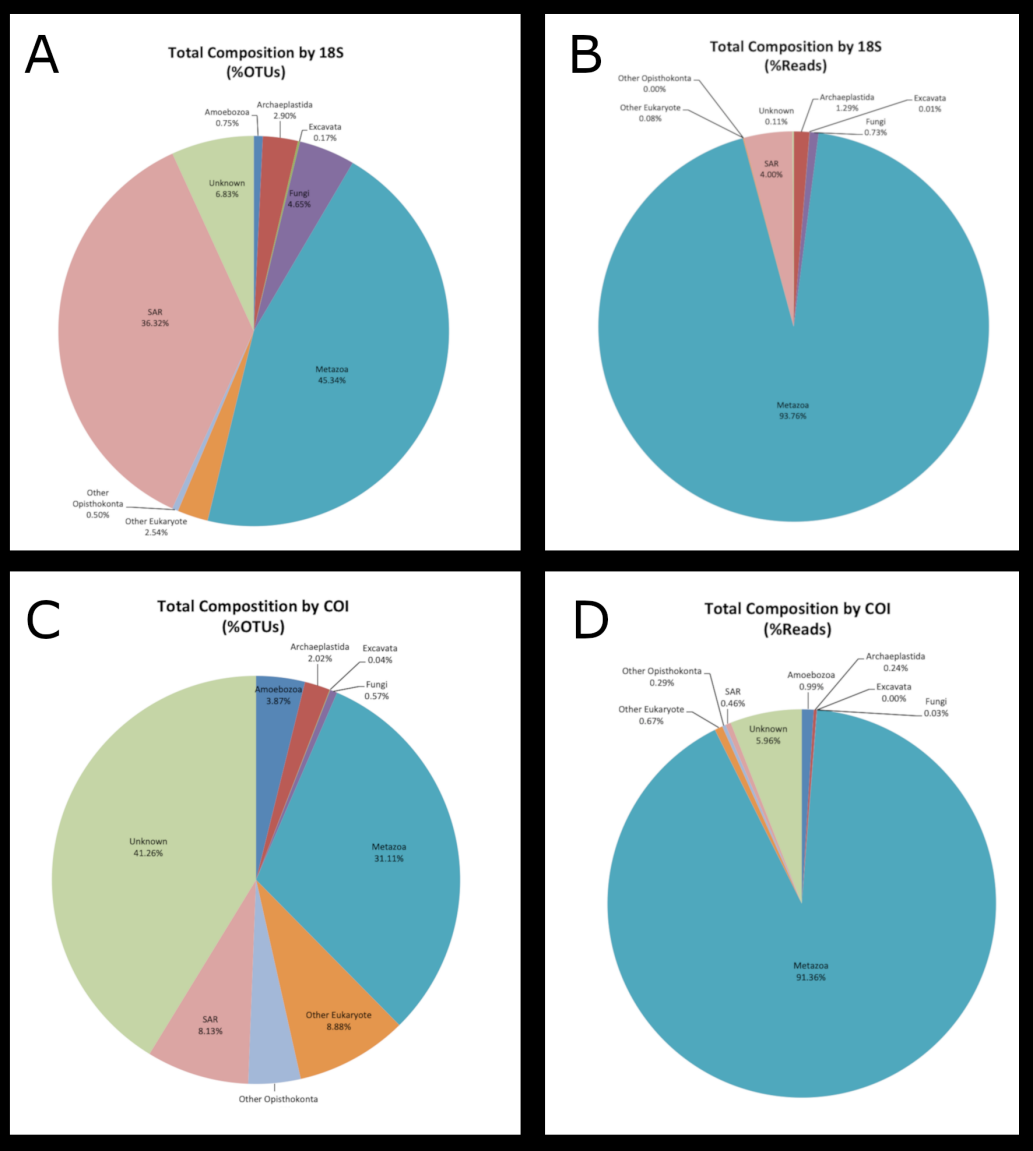
A graphical summary of the higher level composition for all samples combined by (A) the percent of OTUs as determined by 18S sequences; (B) the percent of sequence reads as determined by 18S sequences; (C) the percent of OTUs as determined by COI sequences; (D) the percent of sequence reads as determined by COI sequences.

For the 18S dataset, the majority of OTUs were assigned to either Metazoa (1,639 or 45%) or the SAR superphylum (1,313 or 36%), while 247 (6.8%) were unidentified (Table 2; Fig. 2a,b). For COI, 708 (31%) OTUs were assigned to Metazoa, 185 (8.1%) to the SAR superphylum, and 13 (0.57%) to Fungi, while a large portion of the OTUs (939, 41%) remained unassigned (Table 3; Fig. 2 c,d).

**Figure 3.**
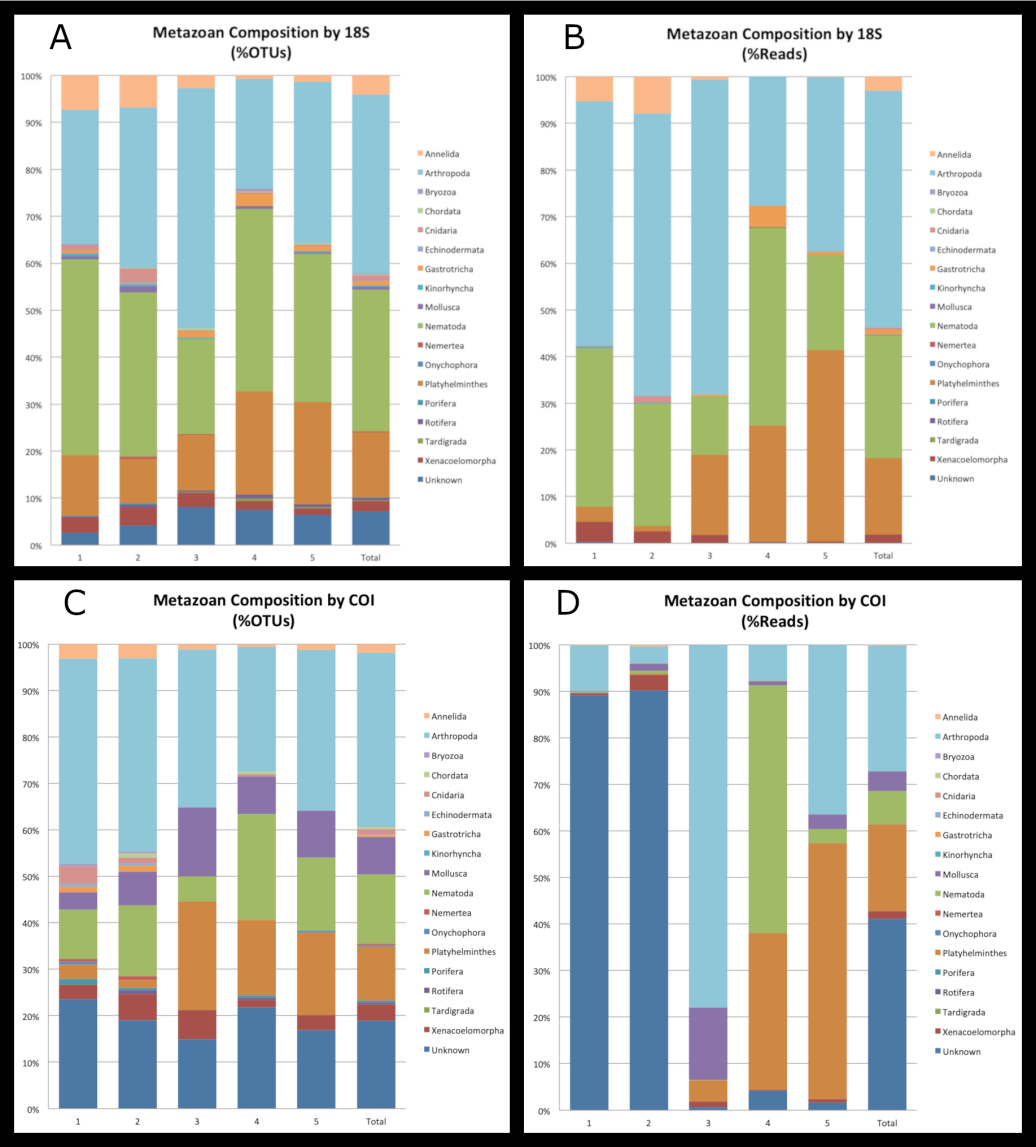
A graphical summary of the Metazoa OTUs identified to phylum level for each sample, as well as all samples combined, as shown by (A) the percent of OTUs based on 18S sequences; (B) the percent of sequence reads based on 18S sequences; (C) the percent of OTUs based on COI sequences; (D) the percent of sequence reads based on COI sequences. 1: Tjärnö, 2: Fiskebäckskil, 3: Halmstad, 4: Kåseberga, 5: Landön. Total: all samples combined.

Tables 4, 5 and Fig. 3 present the OTUs assigned to any Metazoan phylum. For 18S, more than two-thirds of the metazoan diversity based on number of OTUs was split relatively evenly between Arthropoda (624 of 1,639 total OTUs; 38%) and Nematoda (496 OTUs; 30%), with Platyhelminthes accounting for 14% (228 OTUs) and all remaining phyla accounting for only 11% (175 OTUs). A total of 7% (116 OTUs) of the Metazoan OTUs were unable to be identified to phylum level. Such results are consistent with other benthic metabarcoding studies based on the same gene region (Bik et al. 2011, Fonseca et al. 2014, Fonseca et al. 2010, Lallias et al. 2015).

When assessing total number of reads for the 18S dataset, Arthropoda attains more than half the numbers of reads for all of Metazoa (4,621,137 of 9,086,841 total number of reads; 51%), with Nematoda (2391247; 26%) and Platyhelminthes (1,487,606; 16%) following in abundance (Table 4; Fig. 3a,b). All other phyla represent only 6.4% (476,080) of the total number of metazoan reads, with only 0.1% (9,886) remaining unidentified.

For COI, a large amount of the biodiversity could not be assigned to a phylum: approximately 19% (134) based on OTUs and 41% (3,401,317) based on the number of reads. Phyla with high biodiversity in our samples include: Arthropoda with 265 OTUs (37%) across 2,230,455 sequences (27%); Nematoda with 106 OTUs (15%) and 591,410 sequences (7.2%); Platyhelminthes with 82 OTUs (12%) across 1,539,916 sequences (19%); and Mollusca with 57 OTUs (8.1%) and 353,181 sequences (4.3%; Table 5; Fig. 3c,d).

### Community composition at different locations across Sweden

**Figure 4.**
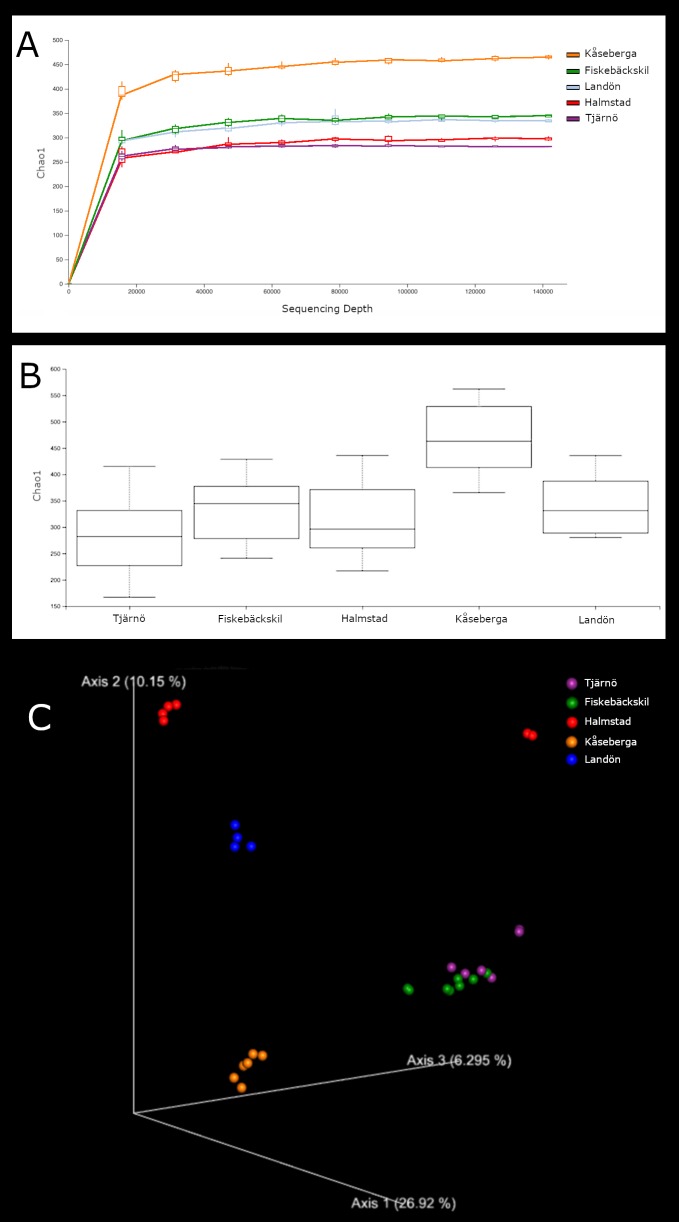
Diversity measurements, based on 18S gene sequences, including (A) rarefaction curves; (B) Chao1 diversity box plots; (C) principal coordinate analysis (PCoA) emperor plots for each sample site.

***18S*.** Fig. 4 shows the alpha diversity rarefaction and box plots for the 18S sequences of each sampling site. Though alpha diversity was consistent across all other sites, Kåseberga had a significantly higher number of OTUs and Chao1 diversity (p < 0.05) than the remaining locations. As can be seen in Table 2, Kåseberga attained much higher numbers of OTUs from every major category except Metazoa. Metazoan diversity of Kåseberga is relatively low, with only 373 OTUs across ~1.5 M sequences. Pairwise ANOSIM analyses (Table 6) indicated that sample composition differed significantly (p ≤ 0.011, R ≥ 0.31) between all sites, excepting Tjärnö and Fiskebäckskil (p = 0.115, R = 0.27). This is illustrated very clearly in the PCoA emperor plot, which shows four distinct clusters representing Halmstad, Kåseberga, Landön and Tjärnö+Fiskebäckskil (Fig. 4c).

The majority of the diversity of every sampling location was assigned either to the SAR supergroup or to Metazoa. Halmstad in particular had much more diversity of Metazoans (619 OTUs across ~ 2.17 M reads) as compared to the other sites (358-465 OTUs across ~ 1.47-2.34 M reads), largely due to the abundance and diversity of the Arthropoda, which was higher here than any other site samples (316 OTUs, ~ 1.47 M reads). Table 4 and Fig. 3 show the Metazoan composition of each location according to amounts of 18S OTUs and reads. Within the metazoan phyla, Arthropoda, Nematoda, Platyhelminthes and Xenacoelomorpha were present in every sample, with Annelida, Cnidaria, Gastrotricha and Rotifera further present at every locale. Such diversity of interstitial taxa is expected in shallow, sandy marine waters.

A number of 18S OTUs were common along the sampled part of the Swedish coast (Table 7). Overall, a total of 37 OTUs were present in every locality, with two of these (one Arthropoda, one Nematoda) present in every single sample. The majority of these shared OTUs (28/37) were identified as Metazoa with the remaining either members of the SAR super assemblage (4), Archaeplastida (3) or Fungi (2).

**Figure 5.**
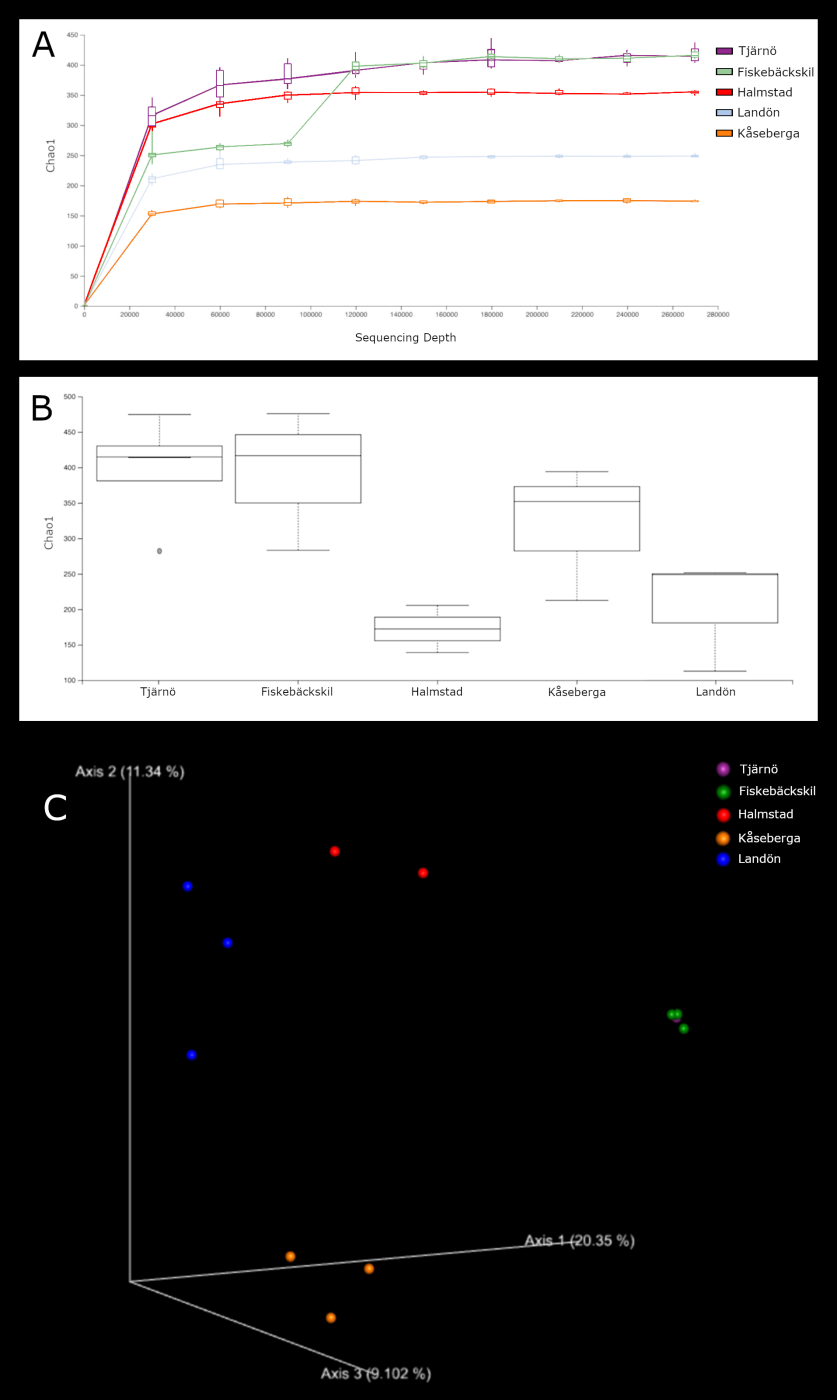
Diversity measurements based on 18S gene sequences, including (A) rarefaction curves; (B) Chao1 diversity box plots; (C) principal coordinate analysis (PCoA) emperor plots for each sample site.

***CO1.*** Fig. 5 gives the alpha diversity rarefaction and box plots based on the CO1 sequences for each sampling site. Although the Chao1 diversities of Tjärnö and Fiskebäckskil were overall slightly higher and that of Halmstad slightly lower, the alpha diversities based on CO1 sequences did not significantly differ between any of the sampling sites (p ≥ 0.05) and results from the pairwise ANOSIM analyses (Table 6) found the composition of each location differed significantly only between Landön and Tjärnö/Fiskebäckskil and Kåseberga and Tjärnö/Fiskebäckskil. As with the 18S results, the PCoA emperor plot found four distinct clusters (Fig. 5c) representing Halmstad, Landön, Kåseberga and Tjärnö+Fiskebäckskil.

OTUs unidentified even to the level of larger assemblages accounted for a large amount of the CO1 datasets for every locale, fully dominating at three of the five locations. Otherwise, the majority of the known diversity was assigned primarily to Metazoa, although fewer metazoan phyla, in general, were represented at each site as compared to the 18S dataset. Of the metazoan phyla represented in the CO1 datasets, Arthropoda, Mollusca, Nematoda and Platyhelminthes were present in every sample, with Annelida and Xenacoelomorpha present at every locale (Table 5; Fig. 3c,d).

Ten CO1 OTUs were present in every location sampled, of which eight were identified as Metazoa (5 Arthropoda, 2 Platyhelminthes, 1 Mollusca) with the remaining two unable to be identified (Table 7). One Platyhelminthes OTU (further identified as a Proseriate) was recorded from every sample.

### Species identification within taxa of Interest

***Acoela***. All but two of the 37 18S-based OTUs initially assigned to Acoela could be positively identified to species level following the phylogeny-based taxonomy assessment (Table 8). Overall, 15 different species of Acoela were identified from the samples, with multiple OTU placements occurring for seven species (2 each for *Anaperus tvaerminnensis*, *Eumecynostomum macrobursalium*, *Mecynostomum lutheri* and *Philactinoposthia* sp. 3; 3 for *Arachaphanostoma* sp. 1; 7 for *Arachaphanostoma agile* and 9 total OTUs identified as *Arachaphostoma macrospiriferum*). The remaining two OTUs were each placed with accumulated likelihoods above 0.90 in a monophyletic clade representing a single genus (*Arachaphanostoma* and *Mecynostomum*).

For COI, 20 of the 25 Acoela OTUs were identified to individual species level (Table 9), with three additional OTUs placed within a single genus clade (*Philactinoposthia*). Of the remaining two, one was identified to family level only (Mecynostomidae) and the other was unable to be placed within a single monophyletic clade with high cumulative likelihood. Overall, five species (*Arachaphanostoma agile*, *Arachaphanostoma macrospiriferum*, *Arachaphanostoma* sp. 1, *Paedomecynostomum bruneum* and *Philactinoposthia* sp. 3) were represented, all with multiple OTU assignments per species.

***Gastrotricha***. For 18S sequences, 10 of the 16 OTUs assigned to Gastrotricha were identified to the species level, with eight OTUs being placed with high likelihood as *Tubanella cornuta* and the remaining two OTUs as *Macrosdasys* sp. 2 and *Halichaetonotus paradoxus* (Table 8). Four OTUs were placed with high cumulative likelihood in single-genus clades (one each in *Chaetonotus* and *Paraturbanella*; two in *Halichaetonotus*) and one OTU was placed within a clade representing a monophyletic family (Turbanellidae). The last OTU could only be assigned to the order Chaetonotida.

Only three COI sequences were assigned to the phylum Gastrotricha and none could be identified to species level (Table 9).

***Macrostomorpha***. Fourteen of the 228 18S-based OTUs initially assigned to Platyhelminthes were identified as belonging to Macrostomorpha and, from these, four could be identified to species level (one OTU each *Dolichomacrostomum uniporum* and *Psammomacrostomum* sp. 1; two OTUs placed as *Microstomum crildensis*), with an additional eight placed in single-genus clades (*Macrostomum*, *Microstomum*). The remaining two OTUs could not be placed within a single, lower-level monophyletic clade (Table 8).

Only two of the 82 OTUs based on COI sequences were identified as Macrostomorpha. One was identified as *Bradynectes sterreri*, while the other was placed in a clade of *Microstomum* (Table 9).

***Rhabdocoela***. The majority (141 of 228) of the Platyhelminthes 18S OTUs were identified as Rhabdoceola. Of these, 78 were identified to species and 21 were placed within single-genus clades, with the remaining 42 only able to be identified to family or higher taxonomic levels (Table 8). Nineteen total species were represented, with the highest number of OTUs (19) placed with high likelihood as *Schizorhynchoides caniculatus* and 10 and 9 OTUs placed as *Placorhynchus octaculeatus* and *Cicerina tetradactyla*, respectively.

For COI, 29 of the 82 OTUs were identified as Rhabdocoela, and all of these were placed with high likelihood as a single species (Table 9). All but one of the 29 OTUs were identified as *Austrorhynchus pacificus*. The last was placed as *Carcharodorhynchus* sp. 24.

## Discussion

### General Notes

This study supplements previous metabarcoding and traditional surveys of the Swedish coastal fauna. Results of both loci found high abundances of Arthropoda, Nematode and Platyhelminthes, consistent with metabarcoding studies utilising the same gene regions (Bik et al. 2011, Fonseca et al. 2014, Fonseca et al. 2010, Haenel et al. 2017, Lallias et al. 2015). Additionally, numerous other interstitial taxa such as Annelida, Gastrotricha and Rotifera were present and reflect the diversity patterns expected of shallow, sandy sediments.

While results between COI and 18S genes were proportionally consistent, there were nevertheless some discrepancies. Primer choice is well known to influence results in a substantial manner. Primer bias driven by mismatches with their target has been shown to skew the relative abundance of amplified DNA from mock communities and, in certain cases, prevent rare species detection (Elbrecht and Leese 2015, Piñol et al. 2015). The COI gene is limited in usefulness because the lack of conserved primer binding sites makes it challenging to design primers that amplify consistently across taxa. The primer pair in this study was designed to amplify a broad range of invertebrate taxa (Haenel et al. 2017) with some emphasis on Platyhelminthes. It is maybe for this reason that smaller phyla, such as Kinorhynchia and Tardigrada, were not evident in the COI dataset.

However, the largest discrepancy in results from the 18S and COI datasets was between the amounts of OTUs that remained unassigned even to higher-level taxa. While the unidentified OTUs for the 18S sequences remained below 10%, nearly half (41%) were unplaced for COI. COI remains the most common DNA marker for animals, but it is less well-used with non-metazoan organisms (Hebert et al. 2003, Leray et al. 2013). There are gaps even within Metazoa, especially in less well-studied taxa, and the Genbank database still lacks COI references for numerous families of marine invertebrates (Curry et al. 2018). Thus, unassigned OTUs may be primarily attributed to the incompleteness of the COI database, although COI barcode misidentifications in GenBank or methodological artifacts (e.g. PCR and sequencing errors or amplification of pseudogenes) also potentially contribute (Bucklin et al. 2016). As the OTUs were assigned to phylum based on an 80% similarity cutoff and COI is most appropriate for higher resolution identifications (Erpenbeck et al. 2006), it is not surprising that a large number of the COI OTUs were unable to be assigned to even a phylum, and our results are consistent with or better than previous metabarcoding studies (e.g. Cowart et al. 2015, Haenel et al. 2017, Leray and Knowlton 2015).

Finally, there were large discrepancies between the number of reads of a particular taxa and the number of OTUs assigned to that taxon. This can clearly be seen when looking at the 18S results (Tables 4, 5; Fig. 3), where Arthropoda and Nematoda attained fairly similar numbers of OTUs (624 or 38%, and 496 or 30%, respectively) but where Arthropoda almost doubled Nematoda in number of reads (4,621,137 or 51% compared with 2,391,247 or 26%). The influence of specimen biomass on sequence read abundance has been previously examined (Elbrecht and Leese 2015, Elbrecht et al. 2017, Thomsen et al. 2016) and a distinct positive correlation between the number of MiSeq reads and the size of an individual animal was found, such that much larger specimens were over-represented in metabarcoding studies when extracted in bulk together with smaller organisms. It may be, then, that taxa composed of smaller individuals are actually under-represented in the data (but see also Lim et al. 2016).

#### Community Composition at Five Locales

This study provides a snapshot of the meiofauna at five different loacations on the Swedish coast. We detected significant community composition differences amongst Locales at higher taxonomic levels, suggesting that metabarcoding even to this small extent can be useful for describing coarse level biodiversity trends. Rarefaction curves for both 18S (Fig. 4a) and COI (Fig. 5a) indicated that sequence variation within the samples was exhaustively captured. For 18S, the asymptote was reached after 20000 reads with each sample yielding between 358 and 619 OTUs and a total of 1639 metazoan OTUs for our five sample sites. Haenel et al. (2017) investigated the effects of sampling methods and primer choice on estimates of Swedish marine meiofauna. While they found lower sublittoral diversity, 266 OTUs in sublittoral sand samples from a single location, they also did not reach the asymptote after 20000 reads of 18S sequences. The alpha diversity of meiofauna along the Swedish west coast is high from a global perspective. The five sites sampled in this study yielded a higher number of OTUs than that detected by Fonseca et al. (2014) when using 18S markers to metabarcode samples from 23 littoral locations in the UK and mainland Europe or by Leasi et al. (2018) in 19 sites at sandy tropical beaches.

Results from the beta analyses identified four statistically-supported clusters corresponding to four distinct and diverse communities, with samples from Tjärnö and Fiskebäckskil grouping together to form a single cluster. The most strikingly different locale was Kåseberga, which unlike all other locations, was dominated by OTUs identified as belonging to the SAR super assemblage (549 18S OTUs) instead of to Metazoa (373 18S OTUs). Indeed, compared to the other Locales, Kåseberga had a much higher number of 18S OTUs from every major category except Metazoa, for which the diversity was comparatively low (Table 2). Further, though the overall biodiversity profiles within Metazoa were driven by commonly occurring taxa, several phyla (e.g. Bryozoa, Cnidaria, Echinodermata, Porifera, Nemertea) occurred predominantly or uniquely within one or two Locales (Tables 4, 5).

Alternatively, a number of 18S OTUs and COI OTUs displayed broad geographic distributions and were present at every locality. Amongst the widely-distributed Metazoa taxa, the two COI OTUs identified as Platyhelminthes (one Proseriate, one otherwise unidentified flatworm) were perhaps the most unexpected, since free-living Platyhelminthes species are thought to have limited dispersal abilities (Palmer 1988). However, without further identification, more should not be inferred at this time.

#### Taxa of Interest

A phylogeny-based taxonomy approach was used to identify OTUs that were preliminarily assigned to four meiofaunal taxa: Acoela, Gastrotricha, Macrostomorpha and Rhabdocoela. These four taxa of interest were selected because their biodiversity within the Swedish littoral sands is arguably well-known compared to most places worldwide. Each has been the focus of previous taxonomic research (e.g. Boaden 1960, Karling 1963, Karling 1974, Luther 1962, Luther 1963, Van Steenkiste et al. 2013, Westblad 1948, Westblad 1954), as well as modern surveys funded by ArtDatabanken and the STI (e.g. Atherton and Jondelius 2019, Kånneby et al. 2012, Kånneby et al. 2013, Larsson and Willems 2010) and DNA sequences of numerous Swedish species are available for each on GenBank. Yet, despite previous efforts, our results found OTUs from all four taxa of interest that could not be identified to species level, suggesting the possibility of new, unknown species within Sweden still remains.

***Acoela***. The reference alignment and subsequent tree used to identify OTUs of Acoela included a total of 343 18S and 185 COI sequences, with numerous new, unpublished sequences and sequences specifically attained from Swedish specimens collected over 25 years. Out of all four taxa of interest, the reference database for Acoela was the most complete and most targeted towards the present study. As such, it is unsurprising that so many of the 18S and COI OTUs could be identified to species level and it demonstrates the potential of the metabarcoding technique to monitor species, map their distributions and otherwise be of use in biodiversity surveys when a comprehensive reference database is available.

It is of particular interest to note, then, that despite the high amounts of reference data, there were OTUs that could not be identified to species level. The unidentified species of *Arachaphanostoma* and *Mecynostomum* based on the 18S gene could potentially be attributed to the fact that 18S does not necessarily differentiate between closely-related species (Tang et al. 2012). *Archaphanostoma occulta* and *A. sublitorallis*, for instance, have identical 18S gene sequences, though they are clearly distinguishable through 28S and COI gene sequences, as well as morphologically (Kånneby et al. 2015). Indeed, close examination of the 18S reference alignment shows other examples where the ~ 370 bp gene region targeted is identical in closely-related species (e.g. *Eumecynostomum flavescens* and *E. westbladi*; *Mecynostomum predatum*, *M. haplovarium* and *M. auritum*; *Proporus carolinensis* and *P. brochii*; Suppl. material 4). However, the COI gene is known to be highly variable between species and is commonly used to distinguish species of Acoela (Kånneby et al. 2015), as well as other meiofauna (e.g. Anslan and Tedersoo 2015, Atherton and Jondelius 2019, Kerbl et al. 2018), so a COI-based OTU failing to match any species would most likely occur only if that OTU represents a species which is not present in the reference database. The five unidentified OTUs based on COI sequences indicate that the Swedish acoel fauna is still incompletely known.

There were several occasions where multiple OTUs were identified as the same species of Acoela. The DADA2 pipeline of QIIME2 clusters OTUs from Exact Sequence Variants (ESV), such that each sequence haplotype is a different OTU (i.e. even a single nucleotide difference makes a new OTU) and assigning multiple OTUs to a single species may simply reflect the intraspecific genetic variation of that species. Every one of the five species of Acoela identified in this study included multiple OTUs based on the COI gene, demonstrating both the expected variability of this gene, as well as the ability of the metabarcoding method and phylogenetic placement technique to capture and assess that variability.

However as previously stated, the 18S locus, though useful for discerning deeper phylogenetic relationships (e.g. Creer et al. 2010, Holterman et al. 2006, Mallatt et al. 2004), may have limited ability to discriminate closely-related or cryptic species (Tang et al. 2012). Little or no intraspecific variation within the 18S genes of acoel species has been measured (Kånneby et al. 2015) and was expected, yet multiple 18S OTUs identified as the same species occurred seven times for Acoela, with as many as seven and nine different OTUs identified as *Arachaphanostoma agile* and *Arachaphostoma macrospiriferum*, respectively. Even if taking a conservative approach and attributing differences in OTU sequences to unusually abundant intraspecific variation or potential sequencing error, such differences should still be limited to only a few bp (Pfeiffer et al. 2018), but larger than expected variability nevertheless occurred in 18S sequences of, for instance, OTUs assigned to *Mecynostomum lutheri*, which differed by 15 bp (4.29%) and those assigned to *Eumecynostomum macrobursalium*, which differed by 19 bp (5.97%, Suppl. material 20). Further, though the majority of the nine OTUs assigned to *Archaphanostomum macrospiriferum* differed from each other by 1-3 bp, one OTU differed from the others by up to 35 bp (9.44%), on par with the number of differences between it and OTUs assigned to *A. agile* and higher than the number of differences, for instance, between OTUs assigned to *A. ylvae* and *A. fontaneti* (13-14 bp). The presence of COI OTUs that could not be assigned to any species as well as a set of 18S OTUs assigned to the same nominal species despite high sequence divergences indicates the presence of hitherto undetected diversity in the Swedish acoel fauna.

A total of 48 species of Acoela (41 described and 7 undescribed species) collected from Sweden were represented in the 18S reference alignment (currently 66 species of Acoela are known from Sweden, with the majority—63/66—recorded from the west coast; Kånneby et al. 2015). Of the 48 species represented, 25 (20 described, 5 undescribed) are known to occur at a comparable depth and habitat (interstitial sand, sublittoral ≤ 20 m depth) as the collected samples and thus may be reasonably expected to be present in the metabarcoding results. In total, 12 of these species (including 31 total OTUs) were detected in our samples (Table 8). Four other species of Acoela previously unrecorded from Sweden were also identified, including *Actinoposthia* sp. 8 (reference sequence collected from Helgoland), *Paramecynostomum* sp UJ0853 (New Caledonia), *Paraproporus* sp. 3 (Italy) and *Paratomella unichaeta* (unknown location).

Sites 1-3 on the west coast showed a much higher acoel 18S biodiversity, each having between 15-18 OTUs (9 or 10 species), as compared to Sites 4 and 5 along the southern coast (7 and 5 OTUs/species, respectively; Table 8). However, despite the high diversity, Sites 1 and 2 together were composed almost entirely of OTUs identified as species already known to Sweden, with only a single potential new record (OTU from Site 1 identified as *Paramecynostomum* sp. UJ08-53), a somewhat unsurprising result given the high degree of previous sampling efforts that have occurred at and near the marine labs at Kristineberg and Tjärnö. Three of the four OTUs identified as species not previously known from Sweden as well as both OTUs that could not definitively be identified were found only in the three less-well-studied southern Locales. The present study is limited in scope and the patterns are tentative, but the results from the 18S sequences indicate that the coast near Site 3/Halmstad, may represent a particularly good area for future sampling efforts, attaining both high amounts of overall acoel biodiversity (18 OTUs/9 species), as well as real potential for new species.

***Gastrotricha***. Gastrotricha of Sweden was relatively-recently surveyed (Curini-Galletti et al. 2012, Kånneby 2011, Kånneby et al. 2009, Kånneby et al. 2012, Kånneby et al. 2013, Todaro et al. 2011, Willems et al. 2009) and records, including some DNA sequences, were presented and published from each location. Currently, 46 species of Gastrotricha are known from Sweden, of which 35 inhabit marine or brackish environments. The 18S reference alignment included representatives from 19 of these 35 marine species (+15 of the 21 species known only from freshwaters), but only three species total, two described (*Halichaetonotus paradoxus*, *Turbanella cornuta*) and one undescribed (*Macrodasys* sp. 2), known from Sweden, were identified from the OTUs assigned to Gastrotricha. Six additional OTUs were found but could not be assigned to any of the Gastrotricha species in the reference alignment. As with Acoela, none the six unidentified OTUs of Gastrotricha was found in Sites 1 and 2, reflecting again how well-studied these sites are compared the southern coast.

Kieneke et al. (2012) tested the genetic variation of the 18S and COI gene in four species of *Turbanella* collected from the Baltic and North Seas, including from two locations on the southern Swedish coast. They found six distinct genetic clusters of *T. cornuta* with much larger between-group genetic distances (p-distance 12.1-18.8%) than within-group distances (maximum 1.5%) for the COI locus. Though only from a single gene, the results nonetheless reflect some potential for cryptic species. Our results further corroborate such potential, with eight OTUs based on 18S sequences all being identified as *Turbanella cornuta*. Five of the eight OTUs differed by no more than 5 bp from each other, as well as the three *T. cornuta* reference sequences, but the other two OTUs differed by 15 to 18 bp. At the very least, as with Acoela, our results suggest that there remains potentially undiscovered species of Gastrotricha within the littoral sands of Sweden.

***Macrostomorpha***. There have been relative few taxonomic studies of macrostomorphs in Sweden apart from a report by Westblad (1953) and a more recent series on marine and freshwater Swedish *Microstomum* (Atherton and Jondelius 2018a, Atherton and Jondelius 2018b, Atherton and Jondelius 2019). Nevertheless, the majority of the OTUs identified as belonging to this group of flatworms could be identified at least to genus level and records are overall consistent with that which has been previously reported. The 18S gene has been demonstrated to be poor at distinguishing between closely-related macrostomorph species (Atherton and Jondelius 2018b), which may be the most likely explanation for the inconclusive identification of so many single-genus OTUs; however, the unidentified Microstomidae OTU based on the COI locus suggests some—albeit limited—missed biodiversity.

***Rhabdocoela***. A little more than half (78 of 141) of the Rhabdocoela OTUs based on 18S data were identified to species level with a further 21 OTUs identified to a single genus. Most of the species (15 of 19) were identified from multiple OTUs and many of these (12 of 15) were collected from more than two distinct locations. The almost ubiquitous widespread geographic ranges and high genetic 18S diversity of the identified species suggests lots of untapped potential for new diversity within this group.

Juxtaposing the relatively well-curated reference database of Acoela, the Rhabdoceola COI reference database was clearly not sufficient for accurate OTU identification. Free-living Rhabdocoela have been recorded from the littoral and sublittoral waters of Sweden (Curini-Galletti et al. 2012, Karling 1974, Van Steenkiste et al. 2013, Willems et al. 2007, Willems et al. 2009), but very few Rhabdocoela COI sequences have been submitted to Genbank (only 43 at time of writing with no sequences of Swedish specimens). For COI, 28 of the 29 OTUs were identified as *Austrorhynchus pacificus*, while the last placed as *Carcharodorhynchus* sp. 2, but further inspection showed that none of these OTUs matched the *Austrorhynchus pacificus* COI reference sequence by over 90%. Undoubtedly, such results are more likely due to the paucity of the COI reference data than a true identification. Metabarcoding-based species identification requires a reference database of DNA sequences that is taxonomically complete and geographically comprehensive for each species and gene region (Bucklin et al. 2016) and such is currently severely lacking for this group of animals.

## Conclusions

This study contributes to the growing body of research surrounding the methodology and applications of metabarcoding. Here, Illumina MiSeq technology was utilised to examine the metazoan community composition at five locations along the Swedish coastline and to assess the biodiversity of four meiofaunal taxa therein.

Our results provide a snapshot of the meiofauna communities in the sampled localities. It is evident that metabarcoding is an effective and efficient method for assessing biodiversity, but it is contingent on the availability of a comprehensive reference database and different representations of genes within public databases can thus affect the quality of the results. Of the four taxa examined in detail, Acoela had the most complete database. Consequently, we were able to identify nearly all OTUs initially designated as acoels to the species level. We can also conclude that unknown acoel species still exist even within the most well-studied parts of Sweden. In juxtaposition, the limited depth of the Rhabdocoela COI reference database meant that even though the rhabdocoel fauna of the sampled area has been extensively studied in the past using traditional methods, metabarcoding of the COI gene failed to provide any further information beyond a basic and ambiguous OTU count.

The differing data and results between 18S and COI also underline the importance of using multiple markers in biodiversity assessments. Within each of Acoela, Gastrotricha, Macrostomorpha and Rhabdocoela, our results showed numerous instances of COI OTUs that could not be identified as a single species, as well as multiple 18S OTUs assigned to a single species. Both instances are interpreted as potential evidence of new species and, taken together, suggests that knowledge of meiofaunal biodiversity is yet incomplete, even in those areas where taxa can be considered best known.

## Supplementary Material

7766C46B-FEDD-5625-94FB-201B8BF75BEA 10.3897/BDJ.8.e51813.suppl1 Supplementary material 1 Supplemental Data 1 Data type Primer Information Brief description Supplemental Data 1: Primers and Protocols for Nested PCR. For the primers, amplicon specific sequences are in bold, Illumina overhang sequences are in red and Illumina adapter sequences are in blue. The Green inserts for primers of PCR step 2 represent the location of the 6 bp Nextera XT i5 and i7 indices (Illumina, catalog FC-131-1001). 18S primers were from Haenel et al. (2017). File: oo_383147.xlsx https://binary.pensoft.net/file/383147 Atherton, S. and Jondelius, U.

759A07AC-F3F6-5083-B53C-D28C0F6CF6F2 10.3897/BDJ.8.e51813.suppl2 Supplementary material 2 Supplemental Data 2 Data type Script Examples Brief description Supplemental Data 2: Script Examples. Includes the scripts used to import the demultiplexed data from SciLifeLab (Stockholm, Sweden) into Qiime2, perform the quality control and chimera removal with DADA2 and perform the alpha rarefaction and other diversity measurements (Principal coordinate analyses, Analysis of Similarities etc.), as well as the scripts used for preliminary OTU identifications with SILVA release 128 and the alignment-based taxonomy assignments of taxa of interest. File: oo_399883.txt https://binary.pensoft.net/file/399883 Atherton, S. and Jondelius, U.

B9DA7895-FBB6-535F-A6BA-B852378E2D96 10.3897/BDJ.8.e51813.suppl3 Supplementary material 3 Supplemental Data 3 Data type GenBank Accession Numbers Brief description Supplemental Data 3: A list of all specimens in the reference databases for the four taxa of interest. Includes the Genbank accession numbers and/or specimen reference number, where applicable. File: oo_383150.fasta https://binary.pensoft.net/file/383150 Atherton, S. and Jonelius, U.

444884CE-70CC-5C7C-A9F0-FB3F6E35717B 10.3897/BDJ.8.e51813.suppl4 Supplementary material 4 Supplemental Data 4 Data type DNA Alignment Brief description Supplemental Data 4: Reference alignment for Acoela based on the 18S gene locus. File: oo_383151.fasta https://binary.pensoft.net/file/383151 Atherton, S. and Jondelius, U.

FFC06E0A-33F0-5EC8-8682-A13A974BFC63 10.3897/BDJ.8.e51813.suppl5 Supplementary material 5 Supplemental Data 5 Data type DNA Alignment Brief description Supplemental Data 5: Reference alignment for Acoela based on the CO1 gene locus. File: oo_383152.fasta https://binary.pensoft.net/file/383152 Atherton, S. and Jondelius, U.

8DE50F5D-5AA5-56DD-93BD-4D7F1418880A 10.3897/BDJ.8.e51813.suppl6 Supplementary material 6 Supplemental Data 6 Data type DNA Alignment Brief description Supplemental Data 6: Reference alignment for Gastrotricha based on the 18S gene locus. File: oo_399884.fasta https://binary.pensoft.net/file/399884 Atherton, S. and Jondelius, U.

AEBD6D99-AFAF-570B-B6B3-4C0CCC015009 10.3897/BDJ.8.e51813.suppl7 Supplementary material 7 Supplemental Data 7 Data type DNA Alignment Brief description Supplemental Data 7: Reference alignment for Gastrotricha based on the 18S gene locus. File: oo_399886.fasta https://binary.pensoft.net/file/399886 Atherton, S. and Jondelius, U.

0BC836DA-A6FE-5A48-AFBB-E6A263644693 10.3897/BDJ.8.e51813.suppl8 Supplementary material 8 Supplemental Data 8 Data type DNA Alignment Brief description Supplemental Data 8: Reference alignment for Macrostomorpha based on the 18S gene locus. File: oo_399887.fasta https://binary.pensoft.net/file/399887 Atherton, S. and Jondelius, U.

E7E81BA3-A77F-54E5-9102-807E816618C7 10.3897/BDJ.8.e51813.suppl9 Supplementary material 9 Supplemental Data 9 Data type DNA Alignment Brief description Supplemental Data 9: Reference alignment for Macrostomorpha based on the COI gene locus File: oo_399889.fasta https://binary.pensoft.net/file/399889 Atherton, S. and Jondelius, U.

41E61D07-FB35-5660-8F18-1B8BDC86CEC9 10.3897/BDJ.8.e51813.suppl10 Supplementary material 10 Supplemental Data 10 Data type DNA Alignment Brief description Supplemental Data 10: Reference alignment for Rhabdocoela based on the 18S gene locus. File: oo_399891.fasta https://binary.pensoft.net/file/399891 Atherton, S. and Jondelius, U.

91CD0696-FA5A-527F-88CB-E19D4A00BADC 10.3897/BDJ.8.e51813.suppl11 Supplementary material 11 Supplemental Data 11 Data type DNA Alignment Brief description Supplemental Data 11: Reference alignment for Rhabdocoela based on the COI gene locus. File: oo_399892.fasta https://binary.pensoft.net/file/399892 Atherton, S. and Jondelius, U.

E5E4BA37-2A58-56E2-B315-877AB49B97C7 10.3897/BDJ.8.e51813.suppl12 Supplementary material 12 Supplemental Data 12 Data type Phylogenetic Placement Tree Brief description Supplemental Data 12: Placement tree of Acoela based on the 18S gene locus. File: oo_399893.jplace https://binary.pensoft.net/file/399893 Atherton, S. and Jondelius, U.

F9B5D61C-A80C-5782-BF49-0E2ACB4D4AF8 10.3897/BDJ.8.e51813.suppl13 Supplementary material 13 Supplemental Data 13 Data type Phylogenetic Placement Tree Brief description Supplemental Data 13: Placement tree of Acoela based on the COI gene locus. File: oo_399894.jplace https://binary.pensoft.net/file/399894 Atherton, S. and Jondelius, U.

5F1515C2-13BF-5F7D-B999-42A971EEDB9D 10.3897/BDJ.8.e51813.suppl14 Supplementary material 14 Supplemental Data 14 Data type Phylogenetic Placement Tree Brief description Supplemental Data 14: Placement tree of Gastrotricha based on the 18S gene locus. File: oo_399896.jplace https://binary.pensoft.net/file/399896 Atherton, S. and Jondelius, U.

F8523762-561D-5873-BFC8-5880E6B95266 10.3897/BDJ.8.e51813.suppl15 Supplementary material 15 Supplemental Data 15 Data type Phylogenetic Placement Tree Brief description Supplemental Data 15: Placement tree of Gastrotricha based on the COI gene locus. File: oo_399897.jplace https://binary.pensoft.net/file/399897 Atherton, S. and Jondelius, U.

29C9F05B-0568-5C90-92CC-5DB321CB768F 10.3897/BDJ.8.e51813.suppl16 Supplementary material 16 Supplemental Data 16 Data type Phylogenetic Placement Tree Brief description Supplemental Data 16: Placement tree of Macrostomorpha based on the 18S gene locus. File: oo_399898.jplace https://binary.pensoft.net/file/399898 Atherton, S. and Jondelius, U.

88C976ED-28E8-5362-885D-F0D46FE74EF6 10.3897/BDJ.8.e51813.suppl17 Supplementary material 17 Supplemental Data 17 Data type Phylogenetic Placement Tree Brief description Supplemental Data 17: Placement tree of Macrostomorpha based on the COI gene locus. File: oo_399899.jplace https://binary.pensoft.net/file/399899 Atherton, S. and Jondelius, U.

9E5C429A-BBF0-59DD-A811-93B691B5F9BA 10.3897/BDJ.8.e51813.suppl18 Supplementary material 18 Supplemental Data 18 Data type Phylogenetic Placement Tree Brief description Supplemental Data 18: Placement tree of Rhabdocoela based on the 18S gene locus. File: oo_399900.jplace https://binary.pensoft.net/file/399900 Atherton, S. and Jondelius, U.

DA0D3EE6-5B86-537C-B1F2-4D575F7CBAA1 10.3897/BDJ.8.e51813.suppl19 Supplementary material 19 Supplemental Data 19 Data type Phylogenetic Placement Tree Brief description Supplemental Data 19: Placement tree of Rhabdocoela based on the COI gene locus. File: oo_399901.jplace https://binary.pensoft.net/file/399901 Atherton, S. and Jondelius, U.

7C8704BB-918C-5824-8200-45F90F4E2768 10.3897/BDJ.8.e51813.suppl20 Supplementary material 20 Supplemental Data 20 Data type No. Nucleotide Differences Brief description Supplemental Data 20. Pairwise number of base pair differences between OTUs identified as species of Acoela for the ~370 bp V1-V2 region of the 18S gene sequence. Ambiguous base pairs were removed for each sequence pair. Differences between OTUs identified as the same species are in bold. File: oo_399902.xlsx https://binary.pensoft.net/file/399902 Atherton, S. and Jondelius, U.

## Figures and Tables

**Table 1.** Total number of reads per marker at each sampling location before and throughout the DADA2 quality control, chimera removal and OTU grouping process.

**18S**	**Input**	**Filtered/Denoised**	**Merged**	**Non-Chimeric**	**OTUs**
1. Tjärnö	5837687	2071031	1947662	1418563	750
2. Fiskebäckskil	12355933	4086192	3428908	2414656	1102
3. Halmstad	7751818	2861465	2716815	2238364	946
4. Kåseberga	8978859	3378949	3094138	2076371	1232
5. Landön	6486137	2447217	2273099	1543469	749
TOTAL	41410434	14844854	13460622	9691423	3615
**CO1**	**Input**	**Filtered/Denoised**	**Merged**	**Non-Chimeric**	**OTUs**
1. Tjärnö	2574531	1202237	1118651	1076404	570
2. Fiskebäckskil	6379738	3130397	3019902	2946301	1172
3. Halmstad	3557580	1780771	1754494	1654651	350
4. Kåseberga	3162540	1474187	1445150	1254788	958
5. Landön	4810016	2350865	2303991	2125578	616
TOTAL	20484405	9938457	9642188	9057722	2482

**Table 2.** A summary of the higher-level identification results from each sample based on the 18S gene sequences. The numbers of OTUs and reads of each taxon overall and at each sampling location are listed.

	**Tjärnö**	**Fiskebäckskil**	**Halmstad**	**Kåseberga**	**Landön**	
	**1**	**2**	**3**	**4**	**5**	**Total**
	#**OTUs / #Reads**	#**OTUs / #Reads**	#**OTUs / #Reads**	#**OTUs / #Reads**	#**OTUs / #Reads**	#**OTUs / #Reads**
Amoebozoa	1 / 2	6 / 124	0 / 0	19 / 998	1 / 2	27 / 1126
Archaeplastida	30 / 44634	35 / 29557	22 / 4583	45 / 43967	24 / 2415	105 / 125156
Excavata	0 / 0	2 / 22	0 / 0	4 / 1396	1 / 11	6 / 1429
SAR	201 / 23514	470 / 41770	214 / 51354	549 / 202797	269 / 68025	1313 / 387460
Other Eukaryote	6 / 409	16 / 682	23 / 2927	43 / 2581	32 / 1205	92 / 7804
Metazoa	465 / 1337416	452 / 2339150	619 / 2171876	373 / 1769973	358 / 1468426	1639 / 9086841
Fungi	20 / 11245	31 / 458	38 / 6056	87 / 50992	35 / 1690	168 / 70441
Other Opisthokonta	1 / 4	7 / 115	4 / 57	7 / 116	2 / 15	18 / 307
Unknown	26 / 1339	83 / 2778	26 / 1511	105 / 3551	27 / 1680	247 / 10859
Total	750 / 1418563	1102 / 2414656	946 / 2238364	1232 / 2076371	749 / 1543469	3615 / 9691423

**Table 3.** A summary of the higher-level identification results from each sample, based on the COI gene sequences. The numbers of OTUs and reads of each taxon overall and at each sampling location are listed.

	**Tjärnö**	**Fiskebäckskil**	**Halmstad**	**Kåseberga**	**Landön**	
	**1**	**2**	**3**	**4**	**5**	**Total**
	#**OTUs / #Reads**	#**OTUs / #Reads**	#**OTUs / #Reads**	#**OTUs / #Reads**	#**OTUs / #Reads**	#**OTUs / #Reads**
Amoebozoa	16 / 7819	28 / 2111	11 / 1434	20 / 6587	45 / 71496	88 / 89447
Archaeplastida	14 / 969	21 / 2008	3 / 5437	10 / 3908	12 / 9621	46 / 21943
Excavata	1 / 67	1 / 254	0 / 0	0 / 0	0 / 0	1 / 321
SAR	60 / 10085	106 / 19381	10 / 3564	12 / 1312	41 / 7428	185 / 41770
Other Eukaryote	69 / 8516	104 / 18510	122 / 5430	32 / 5453	44 / 22356	202 / 60265
Metazoa	161 / 1026022	231 / 2663755	94 / 1545893	248 / 2072072	197 / 954152	708 / 8261894
Fungi	1 / 39	4 / 59	0 / 0	5 / 2281	6 / 221	13 / 2600
Other Opisthokonta	13 / 584	21 / 3210	6 / 11218	9 / 1258	57 / 9562	94 / 25832
Unknown	137 / 20211	346 / 233221	134 / 79906	134 / 30889	295 / 174901	939 / 539128
Total	472 / 1074312	862 / 2942509	280 / 1652882	470 / 2123760	697 / 1249737	2276 / 9043200

**Table 4.** A summary of the Metazoan OTUs identified from each sample to phylum level based on the 18S locus sequences. The number of OTUs and reads of each phylum overall and at each sampling location are listed.

	**Tjärnö**	**Fiskebäckskil**	**Halmstad**	**Kåseberga**	**Landön**	
	**1**	**2**	**3**	**4**	**5**	**Total**
	#**OTUs / #Reads**	#**OTUs / #Reads**	#**OTUs / #Reads**	#**OTUs / #Reads**	#**OTUs / #Reads**	#**OTUs / #Reads**
Annelida	34 / 70747	31 / 184790	17 / 14337	3 / 95	5 / 1438	68 / 271407
Arthropoda	133 / 700309	154 / 1416897	316 / 1465985	87 / 488705	123 / 549241	624 / 4621137
Bryozoa	1 / 10	0 / 0	0 / 0	2 / 498	0 / 0	2 / 508
Chordata	0 / 0	1 / 40	3 / 131	1 / 9	1 / 4	4 / 184
Cnidaria	5 / 1181	13 / 31041	2 / 310	1 / 12	1 / 8	18 / 32552
Echinodermata	0 / 0	1 / 55	0 / 0	0 / 0	0 / 0	1 / 55
Gastrotricha	3 / 1439	1 / 592	7 / 8138	10 / 81062	4 / 9654	16 / 100885
Kinorhyncha	3 / 4241	2 / 4131	2 / 321	0 / 0	1 / 59	5 / 8752
Mollusca	3 / 120	6 / 668	0 / 0	2 / 2939	1 / 5	9 / 3732
Nematoda	194 / 454408	158 / 613752	126 / 272220	145 / 751010	113 / 299857	496 / 2391247
Nemertea	0 / 0	2 / 608	1 / 79	0 / 0	0 / 0	2 / 687
Platyhelminthes	60 / 44008	43 / 27391	73 / 372749	82 / 441294	78 / 602164	228 / 1487606
Porifera	1 / 18	1 / 46	0 / 0	0 / 0	0 / 0	2 / 64
Rotifera	1 / 16	3 / 110	2 / 82	3 / 148	2 / 696	8 / 1052
Tardigrada	0 / 0	0 / 0	1 / 2	2 / 50	1 / 38	3 / 90
Xenacoelomorpha	15 / 57360	17 / 58794	19 / 35249	7 / 1790	5 / 3804	37 / 156997
Unknown	12 / 3559	19 / 235	50 / 2273	28 / 2361	23 / 1458	116 / 9886
Total	465 / 1337416	452 / 2339150	619 / 2171876	373 / 1769973	358 / 1468426	1639 / 9086841

**Table 5.** A summary of the Metazoan OTUs identified from each sample to phylum level based on the CO1 locus sequences. The number of OTUs and reads of each phylum overall and at each sampling location are listed.

	**Tjärnö**	**Fiskebäckskil**	**Halmstad**	**Kåseberga**	**Landön**	
	**1**	**2**	**3**	**4**	**5**	**Total**
	#**OTUs / #Reads**	#**OTUs / #Reads**	#**OTUs / #Reads**	#**OTUs / #Reads**	#**OTUs / #Reads**	#**OTUs / #Reads**
Annelida	5 / 1119	7 / 7467	1 / 157	1 / 382	3 / 808	13 / 9933
Arthropoda	71 / 100897	96 / 98117	32 / 1204537	53 / 72619	86 / 754285	265 / 2230455
Bryozoa	1 / 45	1 / 68	0 / 0	0 / 0	0 / 0	1 / 113
Chordata	0 / 0	2 / 16	0 / 0	1 / 807	0 / 0	3 / 823
Cnidaria	6 / 280	3 / 498	0 / 0	1 / 717	0 / 0	8 / 1495
Echinodermata	1 / 34	1 / 44	0 / 0	0 / 0	0 / 0	1 / 78
Gastrotricha	2 / 576	3 / 800	0 / 0	0 / 0	0 / 0	3 / 1376
Mollusca	6 / 173	17 / 39808	14 / 241184	16 / 8113	25 / 63903	57 / 353181
Nematoda	17 / 2366	35 / 17031	5 / 886	45 / 507414	39 / 63713	106 / 591410
Nemertea	1 / 81	2 / 123	0 / 0	0 / 0	0 / 0	2 / 204
Onychophora	1 / 45	0 / 0	0 / 0	0 / 0	1 / 15	2 / 60
Platyhelminthes	5 / 471	4 / 7888	22 / 70161	32 / 321833	44 / 1139563	82 / 1539916
Porifera	2 / 149	1 / 730	0 / 0	1 / 11	0 / 0	3 / 890
Rotifera	0 / 0	2 / 32	0 / 0	1 / 438	0 / 0	3 / 470
Xenacoelomorpha	5 / 4866	13 / 88069	6 / 19537	3 / 1523	8 / 16178	25 / 130173
Unknown	38 / 914920	44 / 2403064	14 / 9431	43 / 40295	42 / 33607	134 / 3401317
Total	161 / 1026022	231 / 2663755	94 / 1545893	197 / 954152	248 / 2072072	708 / 8261894

**Table 6.** Results of the pairwise analysis of similarity (ANOSIM) analyses between each of the sample sites for both 18S and COI gene sequences. Significant p-values (p < 0.05) are in bold.

**Pairwise ANOSIM Results**				
**Gene**	**Group 1**	**Group 2**	**R**	**p-value**
18S	Halmstad	Landön	0.3081	**0.011**
	Halmstad	Kåseberga	1.0000	**0.004**
	Halmstad	Fiskebäckskil	1.0000	**0.002**
	Halmstad	Tjärnö	0.9722	**0.003**
	Landön	Kåseberga	1.0000	**0.005**
	Landön	Fiskebäckskil	1.0000	**0.001**
	Landön	Tjärnö	1.0000	**0.005**
	Kåseberga	Fiskebäckskil	1.0000	**0.001**
	Kåseberga	Tjärnö	1.0000	**0.002**
	Fiskebäckskil	Tjärnö	0.2698	0.115
CO1	Halmstad	Landön	0.6667	0.108
	Halmstad	Kåseberga	1.0000	0.098
	Halmstad	Fiskebäckskil	1.0000	0.063
	Halmstad	Tjärnö	1.0000	0.064
	Landön	Kåseberga	0.7778	0.092
	Landön	Fiskebäckskil	1.0000	**0.032**
	Landön	Tjärnö	1.0000	**0.038**
	Kåseberga	Fiskebäckskil	1.0000	**0.034**
	Kåseberga	Tjärnö	1.0000	**0.034**
	Fiskebäckskil	Tjärnö	-0.1111	0.744

**Table 7.** OTUs of 18S and COI sequences that were present in at least one sample from all five localities. **OTUs that were further present in every sample.

	**OTUs Present in All Sampling Locations**		
**Gene**	**OTU**	**Super Assemblage**	**Phyla (Metazoa)**
18S	90f6a60093b3fdc85ba7a1cb35057073	Archaeplastida	
	bed5cab4de8b0238d337199cbcbee51e	Archaeplastida	
	fdbc082988924f7c1ecd32c8949fc3c1	Archaeplastida	
	22fa8218e35977d76ebdb9aeb305ac6a	Fungi	
	b04cc75c142fdc390a9737cba8b3016e	Fungi	
	eec420685696d90a0d07d87d1110ab7f	Metazoa	Annelida
	4cc10b4ef5359220090acc37ec45e2c8**	Metazoa	Arthropoda
	5279f7673b35821491c9532851458765	Metazoa	Arthropoda
	86fb00a9f505e8b809054f2b77be9c0f	Metazoa	Arthropoda
	c1f41b8fe6a03d71ad363664c628eba3	Metazoa	Arthropoda
	d3a2d62586eef235d87bf5ea32a69eba	Metazoa	Arthropoda
	dc820b776904fb714527a25dd072bf30	Metazoa	Arthropoda
	e8a53be7443a9c3919d5a70bc01c4e33	Metazoa	Arthropoda
	e92edef507628b3d47afa8f055b42c8d	Metazoa	Arthropoda
	1c33f41b42787bd44f84b7928948c040	Metazoa	Nematoda
	39cbb16486c7a5fd5226e28238c4f361	Metazoa	Nematoda
	3d4364653ddb334a4bd6808b5cb1cddb	Metazoa	Nematoda
	5efaede8aefab69c9471fb10ae896a26**	Metazoa	Nematoda
	6308c0b4f2b682e85b3fcceec379c115	Metazoa	Nematoda
	7ad85c5748dc0f24ff0d0cf699142c8a	Metazoa	Nematoda
	845b4bc23a0b60a614338ad5e16752e2	Metazoa	Nematoda
	a3647b4367700bb20211a2a8c9c6d15f	Metazoa	Nematoda
	bd7d86e4c48f271d2b12f3ebe73a6e0a	Metazoa	Nematoda
	bfc135032b34820cc5dcaa27fcb5d7a0	Metazoa	Nematoda
	ea4de89257c0302aa7191d662ac8ca44	Metazoa	Nematoda
	1ec64309427bdf249f924851356d587f	Metazoa	Platyhelminthes
	25726baf15005d4fc2d1e47eb24465db	Metazoa	Platyhelminthes
	525ec3abad4bf5a6324c56330686504f	Metazoa	Platyhelminthes
	c741c7c5a6e916d37a4bf47635bf293d	Metazoa	Platyhelminthes
	dcb254e4577a5beff4921ca44fc3533e	Metazoa	Platyhelminthes
	e41276741e18d4b131ed89b4e11b6285	Metazoa	Platyhelminthes
	e875782ac3f5d26eaa6956dd4cc35685	Metazoa	Platyhelminthes
	1d5357d35317460eb71604b44982a32b	Metazoa	Xenacoelomorpha
	0bd8922559eed4098d5ac4a255ba7871	SAR	
	64e1b3fa6bbd727a7ef9a9a3cd9eda42	SAR	
	9e872c52ece55247a0a784b494fd3fd6	SAR	
	ebd50793368d8f06efadf1e41e92c178	SAR	
CO1	aee5e5584a107329178954de96480088	Metazoa	Arthropoda
	c091b08c6c169ead97f32db984eb9fe8	Metazoa	Arthropoda
	c44fd006d45976d3eca583816e631c4d	Metazoa	Arthropoda
	cc05db8802aad5d02e18dd1f2780ed6c	Metazoa	Arthropoda
	ff8981ce44e0943f8f2fe599e0c7af20	Metazoa	Arthropoda
	f660505d1d7e488403938eb904f64535	Metazoa	Mollusca
	17f8cd824b6985c53286f7fa0045669c**	Metazoa	Platyhelminthes
	ee6c3481fe8b621d9a9ab8975fbb7418	Metazoa	Platyhelminthes
	3059851980624611c4b991bf0271ec64	UNKNOWN	
	0b5b6eeeeaeda4b023588ad575c77045	UNKNOWN	

**Table 8.** Alignment-based taxonomy assignments of taxa of interest based on 18S sequences. Species of Acoela, Gastrotricha, Macrostomorpha and Rhabdocoela that were identified are listed along with the total number of OTUs and number of reads assigned to each species from each sampling locality and overall. Species in bold are identified species with at least one OTU present at more than one sampling location. **Species that were previously found within Sweden.

	**Tjärnö**	**Fiskebäckskil**	**Halmstad**	**Kåseberga**	**Landön**	
	**1**	**2**	**3**	**4**	**5**	**Total**
	#**OTUs / #Reads**	#**OTUs / #Reads**	#**OTUs / #Reads**	#**OTUs / #Reads**	#**OTUs / #Reads**	#**OTUs / #Reads**
** XENACOELOMORPHA **						
*Actinoposthia* sp. 8	0 / 0	0 / 0	1 / 7	0 / 0	0 / 0	1 / 7
Anaperus tvaerminnensis**	0 / 0	2 / 33	1 / 5190	1 / 20	1 / 30	2 / 5273
*Aphanostoma* sp. AWHel19**	1 / 576	0 / 0	0 / 0	0 / 0	0 / 0	1 / 576
Archaphanostoma agile**	4 / 27639	5 / 22978	1 / 466	1 / 20	0 / 0	*7 / 51103*
Archaphanostoma macrospiriferum**	1 / 1772	1 / 688	9 / 24026	1 / 1615	1 / 3711	9 / 31812
Archaphanostoma ylvae**	0 / 0	0 / 0	3 / 4483	1 / 39	1 / 43	3 / 4565
Archaphanostoma fontaneti**	1 / 242	1 / 346	0 / 0	0 / 0	0 / 0	1 / 588
Eumecynostomum macrobursalium**	2 / 25304	2 / 29170	1 / 945	1 / 16	0 / 0	2 / 55435
*Isodiametra* sp. 2**	1 / 92	1 / 1497	0 / 0	0 / 0	0 / 0	1 / 1589
Mecynostomum auritum**	1 / 68	1 / 97	1 / 10	0 / 0	0 / 0	1 / 175
Mecynostomum lutheri**	2 / 683	2 / 1947	1 / 43	0 / 0	0 / 0	2 / 2673
*Paramecynostomum* sp. UJ0853	1 / 10	0 / 0	0 / 0	0 / 0	0 / 0	1 / 10
*Paraproporus* sp. 3	0 / 0	0 / 0	0 / 0	0 / 0	1 / 10	1 / 10
*Paratomella unichaeta*	0 / 0	0 / 0	1 / 79	0 / 0	1 / 10	1 / 89
*Philactinoposthia* sp. 3**	1 / 974	2 / 2038	0 / 0	0 / 0	0 / 0	2 / 3012
Archaphanostoma species	0 / 0	0 / 0	0 / 0	1 / 5	0 / 0	1 / 5
*Mecynostomum* species	0 / 0	0 / 0	0 / 0	1 / 75	0 / 0	1 / 75
** MACROSTOMORPHA **						
Dolichomacrostomum uniporum**	1 / 1160	0 / 0	1 / 23356	1 / 5886	1 / 7690	1 / 38092
Microstomum crildensis**	2 / 906	1 / 1663	0 / 0	1 / 1495	0 / 0	2 / 4064
*Psammomacrostomum* sp. 1**	0 / 0	0 / 0	1 / 1142	1 / 52	1 / 496	1 / 1690
*Macrostomum* species	0 / 0	1 / 318	0 / 0	0 / 0	0 / 0	1 / 318
*Microstomum* species	3 / 3467	6 / 5539	2 / 168	1 / 912	0 / 0	7 / 10086
Dolichomacrostomidae species	0 / 0	0 / 0	0 / 0	0 / 0	1 / 5	1 / 5
Macrostomorpha species	0 / 0	0 / 0	0 / 0	1 / 39	0 / 0	1 / 39
** RHABDOCOELA **						
Brinkmanniella palmata**	1 / 90	4 / 264	0 / 0	0 / 0	0 / 0	4 / 354
*Cheliplana orthocirra*	1 / 2182	1 / 6	1 / 5018	1 / 21891	4 / 63315	4 / 92412
Cicerina tetradactyla**	4 / 6419	1 / 29	5 / 29307	2 / 41527	4 / 86616	9 / 163898
Diascorhynchus serpens**	1 / 2469	0 / 0	1 / 243	2 / 7823	3 / 9383	4 / 19918
Gnathorhynchus inermis**	1 / 1240	0 / 0	3 / 5823	1 / 5471	2 / 21330	4 / 33864
*Litucivis serpens*	0 / 0	0 / 0	0 / 0	0 / 0	1 / 1429	1 / 1429
Odontorhynchus aculeatus**	0 / 0	1 / 21	0 / 0	0 / 0	0 / 0	1 / 21
*Paracicerina laboeica*	2 / 4058	0 / 0	4 / 63699	1 / 25718	1 / 30001	5 / 123476
*Paracrorhynchus* sp. TJ2014	1 / 287	0 / 0	0 / 0	0 / 0	0 / 0	1 / 287
Placorhynchus dimorphis**	1 / 141	0 / 0	0 / 0	1 / 1392	0 / 0	2 / 1533
Placorhynchus octaculeatus**	2 / 751	1 / 10	3 / 480	6 / 41174	2 / 590	10 / 43005
*Prognathorhynchus busheki*	1 / 830	2 / 2011	1 / 446	0 / 0	0 / 0	2 / 3287
Proxenetes quinquespinosus**	2 / 793	1 / 694	1 / 640	0 / 0	0 / 0	2 / 2127
Psammorhynchus tubulipenis**	2 / 314	0 / 0	0 / 0	0 / 0	0 / 0	2 / 314
Ptychopera westbladi**	0 / 0	1 / 15	0 / 0	0 / 0	0 / 0	1 / 15
*Schizorhynchoides caniculatus*	3 / 2407	2 / 55	9 / 72324	3 / 7928	12 / 114977	19 / 197691
*Thylacorhynchus ambronensis*	1 / 162	0 / 0	3 / 5685	0 / 0	1 / 1509	3 / 7356
Uncinorhynchus flavidus**	1 / 223	1 / 372	0 / 0	1 / 214	0 / 0	2 / 809
Zonorhynchus seminascatus**	2 / 50	2 / 63	0 / 0	0 / 0	0 / 0	2 / 113
*Ceratopera* species	0 / 0	0 / 0	0 / 0	1 / 3622	0 / 0	1 / 3622
*Cheliplana* species	2 / 194	0 / 0	2 / 1330	1 / 10	5 / 13949	8 / 15483
*Gnathorhynchus* species	1 / 152	0 / 0	1 / 456	1 / 99	2 / 2021	2 / 2728
*Pogaina* species	1 / 68	1 / 27	0 / 0	0 / 0	0 / 0	1 / 95
*Proxenetes* species	2 / 1672	2 / 2091	0 / 0	0 / 0	0 / 0	3 / 3763
*Thylacorhynchus* species	0 / 0	0 / 0	2 / 3732	2 / 304	3 / 2098	5 / 6134
*Toia* species	1 / 6	1 / 832	0 / 0	0 / 0	0 / 0	1 / 838
Cicerininae species	2 / 11	0 / 0	4 / 17	5 / 34	3 / 1041	13 / 1103
Gnathorhynchidae species	1 / 304	1 / 1264	0 / 0	0 / 0	0 / 0	1 / 1568
Promesostomidae species	0 / 0	0 / 0	2 / 311	3 / 950	3 / 10079	3 / 11340
Schizorhynchidae species	0 / 0	0 / 0	2 / 13	0 / 0	2 / 49	4 / 62
Schizorhynchia species	0 / 0	0 / 0	1 / 3	0 / 0	1 / 3	2 / 6
Thalassotyphloplanida species	3 / 268	3 / 350	0 / 0	0 / 0	2 / 11	8 / 629
Eukalyptorhynchia species	1 / 105	0 / 0	0 / 0	3 / 3636	1 / 37	4 / 3778
Neodalyellida species	0 / 0	0 / 0	1 / 244	0 / 0	0 / 0	1 / 244
Dalytyphloplanida species	1 / 366	1 / 386	0 / 0	0 / 0	0 / 0	1 / 752
Kalyptorhynchia species	1 / 27	0 / 0	0 / 0	0 / 0	0 / 0	1 / 27
Rhabdocoela species	1 / 21	1 / 236	1 / 103	0 / 0	0 / 0	4 / 360
** GASTROTRICHA **						
*Macrodasys* sp2**	1 / 362	1 / 592	0 / 0	0 / 0	0 / 0	1 / 954
Halichaetonotus paradoxus**	0 / 0	0 / 0	1 / 911	1 / 375	0 / 0	1 / 1286
Turbanella cornuta**	2 / 1077	0 / 0	3 / 6495	5 / 79970	1 / 8684	8 / 96226
*Chaetonotus* species	0 / 0	0 / 0	0 / 0	0 / 0	1 / 2	1 / 2
*Halichaetonotus* species	0 / 0	0 / 0	1 / 499	2 / 47	1 / 268	2 / 814
*Paraturbanella* species	0 / 0	0 / 0	0 / 0	1 / 10	0 / 0	1 / 10
Turbanellidae species	0 / 0	0 / 0	1 / 3	0 / 0	0 / 0	1 / 3
Chaetonotida species	0 / 0	0 / 0	1 / 230	1 / 660	1 / 700	1 / 1590

**Table 9.** Alignment-based taxonomy assignments of taxa of interest based on COI sequences. Species of Acoela, Gastrotricha, Macrostomorpha and Rhabdocoela that were identified are listed along with the total number of OTUs and number of reads assigned to each species from each sampling locality and overall. Species in bold are identified species with at least one OTU present at more than one sampling location. **Species that were previously found within Sweden.

	**Tjärnö**	**Fiskebäckskil**	**Halmstad**	**Kåseberga**	**Landön**	
	**1**	**2**	**3**	**4**	**5**	**Total**
	#**OTUs / #Reads**	#**OTUs / #Reads**	#**OTUs / #Reads**	#**OTUs / #Reads**	#**OTUs / #Reads**	#**OTUs / #Reads**
** XENACOELOMORPHA **						
Archaphanastoma agile**	2 / 1676	7 / 56506	0 / 0	0 / 0	0 / 0	7 / 58182
Archaphanastoma macrospiriferum**	0 / 0	0 / 0	1 / 5	0 / 0	3 / 37	3 / 42
Archaphanastoma ylvae**	0 / 0	1 / 12	5 / 19532	1 / 503	1 / 8	5 / 20055
Paedomecynostomum bruneum**	0 / 0	0 / 0	0 / 0	1 / 1017	3 / 16104	3 / 17121
*Philactinoposthia* sp. 3**	0 / 0	2 / 201	0 / 0	0 / 0	0 / 0	2 / 201
*Philactinoposthia* species	1 / 3090	2 / 31277	0 / 0	1 / 3	1 / 29	3 / 34399
Mecynostomidae species	1 / 93	1 / 73	0 / 0	0 / 0	0 / 0	1 / 166
Acoela species	1 / 7	0 / 0	0 / 0	0 / 0	0 / 0	1 / 7
** MACROSTOMORPHA **						
Bradynectes sterreri**	0 / 0	0 / 0	0 / 0	1 / 14	0 / 0	1 / 14
Microstomidae	0 / 0	0 / 0	0 / 0	1 / 8	0 / 0	1 / 8
** RHABDOCOELA **						
*Austrorhynchus pacificus*	1 / 8	1 / 13	13 / 18126	13 / 28311	11 / 62680	28 / 109138
*Carcharodorhynchus* sp. 24	0 / 0	0 / 0	0 / 0	0 / 0	1 / 35	1 / 35
** GASTROTRICHA **						
Chaetonotida species	2 / 576	2 / 794	0 / 0	0 / 0	0 / 0	2 / 1370
Gastrotricha species	0 / 0	1 / 6	0 / 0	0 / 0	0 / 0	1 / 6
